# Case Report: Prenatal diagnosis of fetal tetrasomy 9p initially identified by non-invasive prenatal testing

**DOI:** 10.3389/fgene.2022.1020525

**Published:** 2022-10-31

**Authors:** Jialing Yu, Na Chen, Min Chen, Min Shen, Yeqing Qian, Minyue Dong

**Affiliations:** ^1^ Women’s Hospital, School of Medicine, Zhejiang University, Hangzhou, Zhejiang, China; ^2^ Key Laboratory of Reproductive Genetics Zhejiang University, Ministry of Education, Hangzhou, Zhejiang, China; ^3^ Key Laboratory of Women’s Reproductive Health of Zhejiang Province, Women’s Hospital, School of Medicine, Zhejiang University, Hangzhou, China

**Keywords:** tetrasomy 9p syndrome, NIPT, prenatal diagnosis, CMA, karyotyping

## Abstract

Tetrasomy 9p is a rare syndrome characterized by fetal growth restriction, Dandy-Walker malformation, cardiac anomalies, and facial abnormalities and is discovered by ultrasound during the prenatal examination. Herein, we report a fetus of tetrasomy 9p without obvious phenotypic manifestations during the first trimester that was identified by non-invasive prenatal testing (NIPT). NIPT revealed that the gain of 9p24.3–9p11 that was approximately 46.36 Mb in size. Karyotyping of amniocytes indicated an additional marker in all metaphase. Chromosome microarray and fluorescence *in situ* hybridization on uncultured amniocytes revealed tetrasomic of 9p24.3q13, and that the supernumerary chromosome is a dicentric isochromosome consisted of two copies of the 9p arm. Taken together, it was indicated that the fetal karyotype was 47,XY,+idic (9) (q13), and that multiple techniques are crucial to the prenatal diagnosis.

## Introduction

Tetrasomy 9p (T9p), which was first defined in 1973 (Ghymers et al., 1973), is a rare abnormality typically resulting from a supernumerary isochromosome and mostly documented after birth. The phenotype of T9p varies from fetuses with multiple abnormalities to phenotypically normal adults (Bellil et al., 2020; Shu et al., 2021). Fetuses with T9p usually exhibit abnormal ultrasound findings including facial clefts, fetal growth retardation (FGR), and Dandy-Walker variant (Chen et al., 2007). So far, few cases have been diagnosed prenatally.

Non-invasive prenatal testing (NIPT) is emerging as a robust technique to screen for trisomies in 13, 18, 21, and sex chromosome (Lo et al., 1997; Lo et al., 1998; Song et al., 2013; Yin et al., 2015; Gross et al., 2016). Furthermore, its ability in screening for subchromosomal abnormalities such as Cri-du-chat deletions, 1p36 deletion syndrome, Wolf-Hirschhorn syndrome, Prader-Willi deletions, or Angelman deletions, has been proven as shown by high predictive positive value (Liu et al., 2016; Liang et al., 2019).

In the current investigation, we present a case of non-mosaic T9p that was identified through NIPT and validated by the combination of karyotyping, chromosome microarray (CMA), and fluorescence *in situ* hybridization (FISH).

## Patients and methods

### Case presentation

A healthy 37-years-old pregnant woman, who claimed no family history of genetic abnormalities and previously delivered two healthy boys, was referred to Women’s Hospital, School of medicine, Zhejiang University. She had unremarkable ultrasound screening at 12^th^ week of gestation that revealed a normal nuchal translucency (NT) and the presence of nasal bone, while she received NIPT at the 15^th^ week of gestation because of advanced maternal age. NIPT showed a duplication at 9p24.3p11.2. Amniocentesis was performed at the 18th week of gestation, and the fetal sample was analyzed by karyotyping, CMA, and FISH.

After genetic counseling, the woman and her husband decided to terminate the pregnancy; however, they declined to undergo a fetal MRI and fetal autopsy.

### Non-invasive prenatal testing screening

NIPT procedures, including cell-free DNA extraction, library construction, and next-generation sequencing (NGS) were performed as described previously (Chen et al., 2019). Bioinformatic methods combined with a locally weighted polynomial regression were used to eliminate GC-bias, and a binary hypothesis was performed to obtain a higher accuracy for NIPT detection. Low coverage whole genome sequencing of plasma DNA was carried out on each sample, resulting in 10.19 million unique reads that corresponds to 0.1 × human genome depth. A fetal copy-number analysis (CNV) was performed to detect subchromosomal deletion and duplication, as previously described (Chen et al., 2019).

### DNA extraction

DNA extraction was performed as described previously (Qian et al., 2019). Briefly, 10 ml maternal blood sample was centrifuged at 1,600 g for 10 min. The supernatant was re-centrifuged at 14,000 g for another 10 min. The plasma fraction was aliquoted and stored at −80°C for subsequent NIPT. The amniotic fluid was centrifuged at 1,600 g for 10 min. Fetal DNA from the centrifuged amniotic fluid cells were then extracted with QIAamp DNA Blood Mini Kit (Qiagen, Hilden, Germany).

### Amniocentesis and cytogenetic analysis

Transabdominal amniocentesis was performed under the real-time sonographic guidance. A total of 20 ml of amniotic fluid was withdrawn after discarding the first 2 ml of amniotic fluid (Jindal et al., 2022). Fetal amniocytes were cultured, and GTG-banding was performed according to standard cytogenetic procedures, yielding a 320–400 band level with a resolution of around 10 Mb. Generally, 30 metaphases were counted, and 5 metaphases were analyzed (Jin et al., 2021).

### Chromosome microarray

Genomic DNA was extracted with the GentraPuregene Kit (Qiagen, Germany) from fetal amniotic cells. CytoScan^TM^ HD array (Affymetrix, United States) was used in copy number analysis according to the manufacture’s instructions. The array is characterized by > 2,600,000 CNV markers, including 750,000 SNP probes and >1,900,000 non–polymorphism probes for comprehensive whole genome coverage. Chromosome Analysis Suite (ChAS) software (Affymetrix, United States) was used to visualize and analyze the results. The reporting threshold of the copy number result was set at 500 kb with marker count ≥50 for gains and 200 kb with a marker count of ≥50 for losses. The analysis was based on the GRCh37/hg19 assembly.

### Fluorescence *in situ* hybridization analysis

Amniocytes were quadruple stained with the chromosome 9 subtelomeric p, q, and centromeric probe (Vysis, Downers Grove, IL) and DAPI (Vysis, Downers Grove, IL). The slides were hybridized according to the manufacture’s instruction and counterstained with DAPI, then analyzed by Zeiss Imager A2 microscope (Zeiss, France). Image acquisition was subsequently performed using a CCD camera with Isis (FISH Imaging System, MetaSystems, Germany).

## Results

### Non-invasive prenatal testing results

**FIGURE 1:**
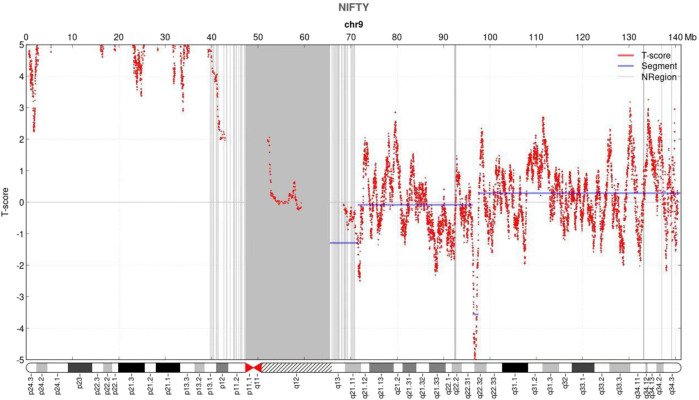
Non-invasive prenatal testing (NIPT) results of fetal chromosome 9. The horizontal axis represents genomic location (Mb), and the vertical axis represents t-score. NIPT revealed an increase in the signal of the p arm of chromosome 9.

NIPT showed negative for other chromosomes expect for the chromosome 9 with a fetal fraction DNA concentration of 10.72%. Additionally, an elevated amount of DNA from the p-arm of chromosome 9 was observed with a mean t-score of 11.528, suggesting a gain of approximately 46.36 Mb that encompassed chromosome bands 9p24.3–9p11 (Figure 1).

### Karyotyping, chromosome microarray, and fluorescence *in situ* hybridization results

**FIGURE 2:**
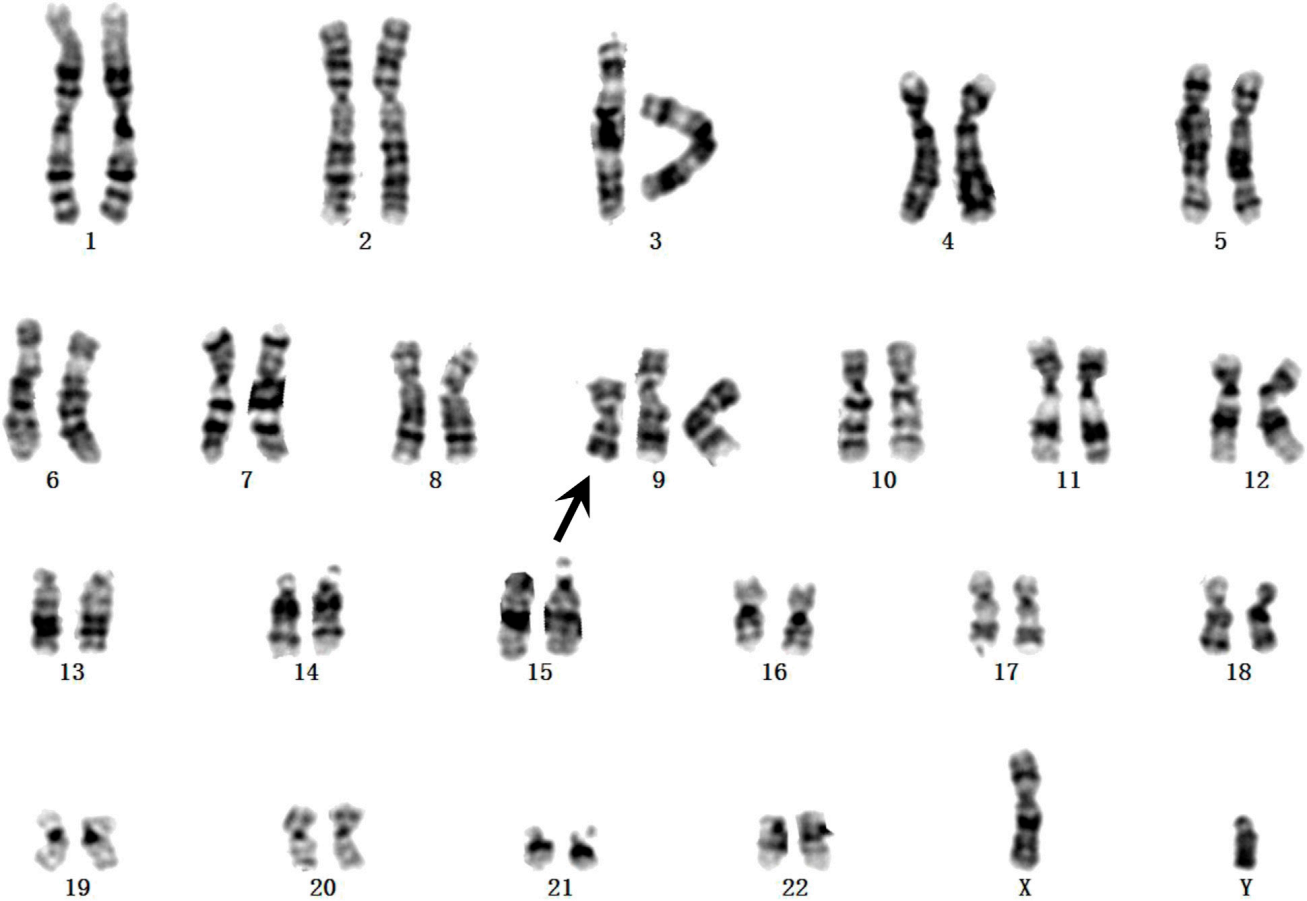
GTG-banding from the amniotic fluid sample. The supernumerary chromosome is shown by black arrow.

**FIGURE 3:**
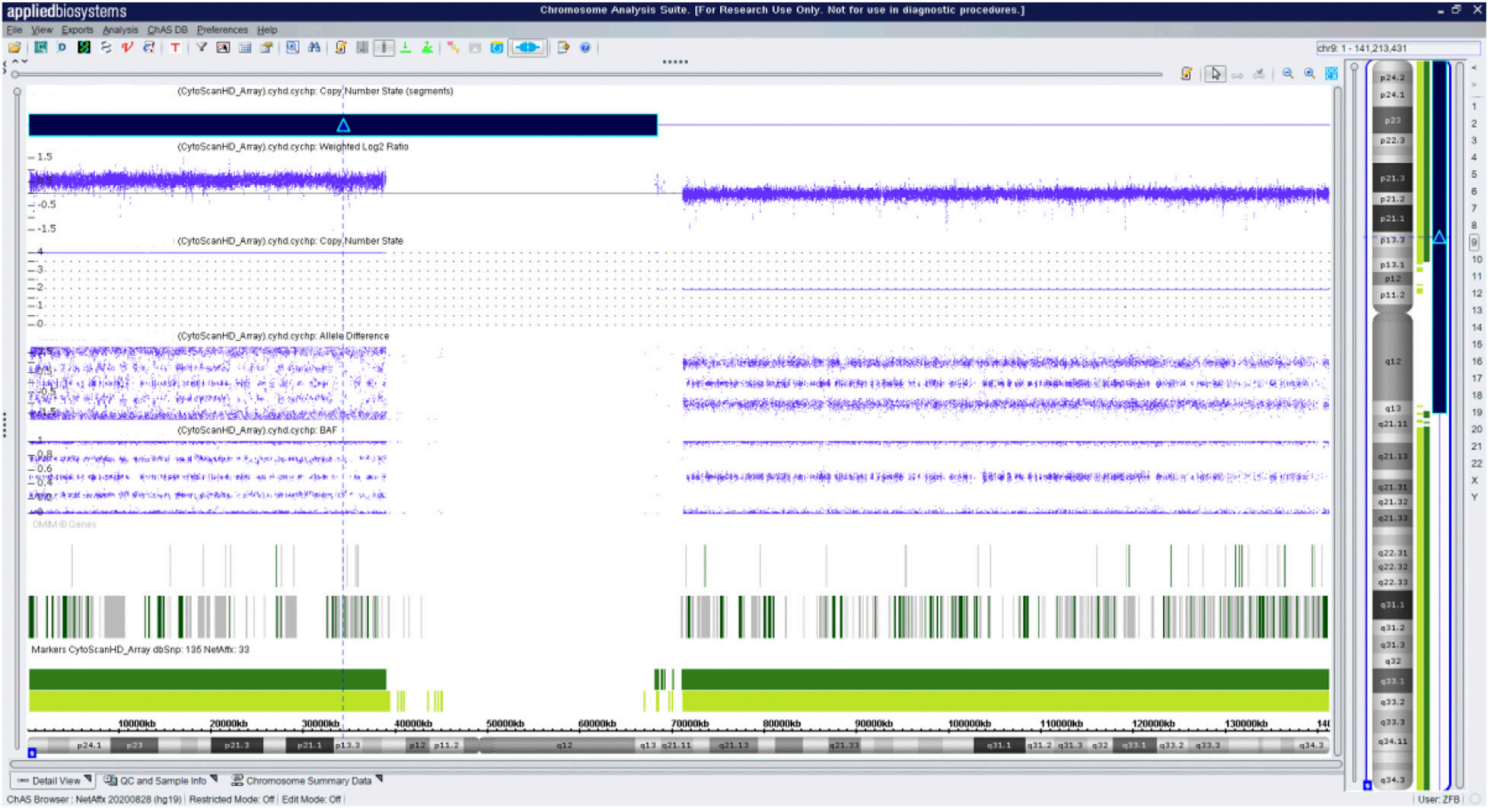
Chromosome microarray on DNA prepared from uncultured amniocytes. Results for chromosome 9 showing gain of the entire p arm and a portion of the q arm.

Fetal karyotyping of the cultured aminocytes showed a male karyotype with the presence of supernumerary marker chromosome (SMC) (30/30) (Figure 2). CMA of the fetal DNA extracted from the uncultured aminocytes revealed a gain of approximately 68.126 Mb spanning from 9pter to 9q13 with a four-fold dose as indicated by five lines in both allele difference and BAF graphics (Figure 3). The array karyotype was: arr [GRCh37] 9p24.3q13 (203862_68330127)x4 (Figure 3).

**FIGURE 4:**
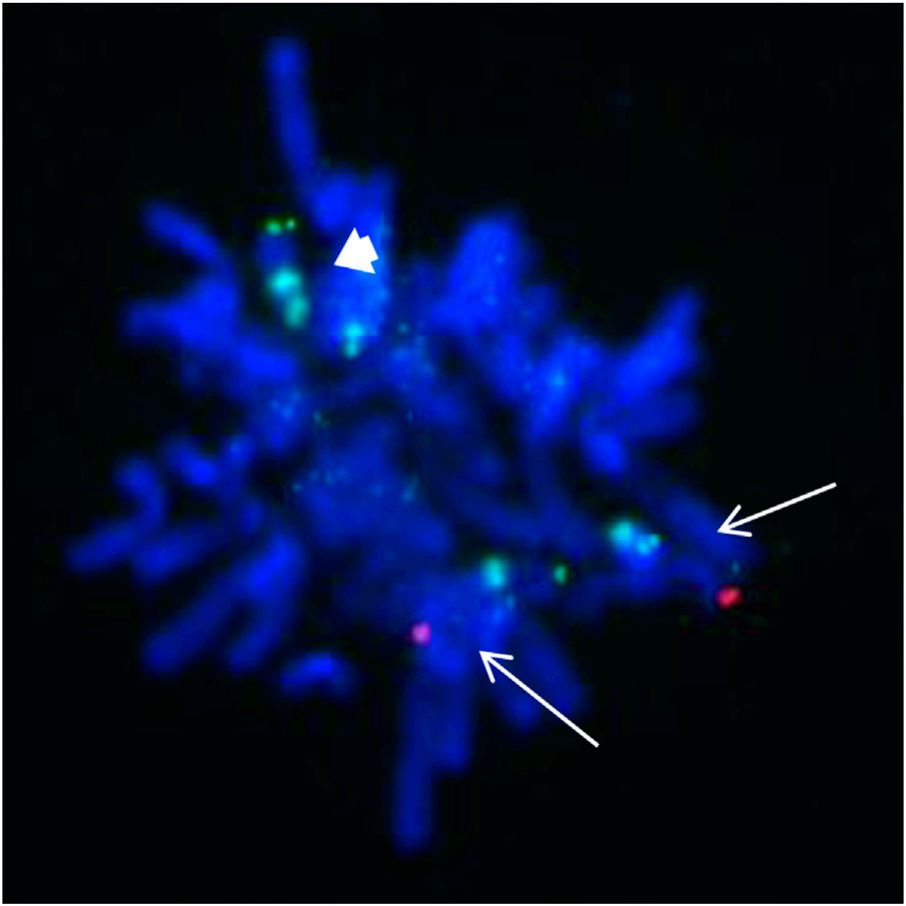
FISH from the uncultured amniocytes. Green signals indicate the subtelomeric part of chromosome 9p; red signals indicate the subtelomeric part of chromosome 9q; aqua signals indicate the centromeic of chromosome 9. Two normal chromosome 9 contain both green and red signals as showed with white arrow. The isodicentric chromosome 9p (bold white arrow) with two green signals and two aqua signals.

These findings suggest that the SMC was a tetrasomy of 9p. The gain of the short arm of chromosome 9 was confirmed by FISH, which was carried out using the chromosome 9 specific subtelomeric p, q, and centromeric probe (Figure 4). FISH showed that the SMC was an isochromosome consisting of two copies of the entire short arm and the heterochromatic region of the long arm of a chromosome 9 with two centromeres. Based on karyotyping, CMA and FISH, the fetal karyotype was 47,XY,+idic (9) (q13).ish idic (9) (q13) (305J7-T7+,D9Z1+,D9Z1+,305J7-T7+).arr [GRCh37] 9p24.3q13 (203862_68330127)x4.

## Discussion

Herein, we reported a prenatal case of tetrasomy 9p that presented without obvious ultrasound anomalies during the first trimester. To the best of our knowledge, our case is the first prenatal case of non-mosaic tetrasomy 9p identified through NIPT.

NIPT may detect CNVs with a size greater 10 Mb with high sensitivity and specificity (Lo et al., 1997; Lo et al., 1998; Song et al., 2013; Yin et al., 2015; Gross et al., 2016). Additionally, it has good performance in detecting known microduplication/microdeletion syndromes (MMS), which are smaller than 10 Mb, such as DiGeorge (93%) and 22q11.22 microduplication (68%) (Liang et al., 2019). Our data showed that NIPT provided quite precise duplication localization which was validated by amniocyte CMA results, suggesting the reliability of NIPT in detecting rare chromosome anomalies such as tetrasomy 9p.

Besides, NIPT can screen MMS before visible abnormalities are revealed on routine prenatal ultrasound examination. Most T9p fetuses were identified through ultrasound findings. Among the 60% of cases reviewed by Vinksel et al. (2019) fetuses with T9p were identified through ultrasound performed during the second and third trimesters. Among the cases reported in the first trimester, increased NT was the most common features, which can be present as an isolated phenotype or accompanied by other anomalies (cleft lip and/or palate, facial dysmorphism and skeletal abnormalities) (Vinksel et al., 2019; Kok Kilic et al., 2022). In our case, only the first trimester ultrasound scan was performed; therefore, other common abnormalities associated with T9p might not have displayed. In another case of mosaic T9p identified through NIPT, the pregnant women presented with a normal NT and nasal bone in the first trimester, while showed isolated persistent left superior vena cava until 25^th^ week gestation ultrasound examination (Wang et al., 2015). Both this case and our case indicate that NIPT is superior to echography in screening for early prenatal abnormalities.

It was suggested that the degree of the mosaicism is associated with the severity of the phenotype of T9p. However, the severity of the phenotype of T9p is not linearly correlated with the mosaic level of the supernumerary chromosome in lymphocyte samples. A case with full tetrasomy 9p in the blood, 65% mosaic in the buccal mucosa, was a health woman who was accidentally identified due to her previous pregnancy with an inv (7) baby (Papoulidis et al., 2012). Among the 8 cases reported with a clinically normal phenotype, the tetrasomic clone ranged from 6% to 100% in peripheral blood lymphocytes, except in five cases that had fertility related issues (5/8) (Papoulidis et al., 2012; Bellil et al., 2020; Shu et al., 2021).

One might speculate that the tissue-limited mosaicism is related to severity of the phenotype of T9p. Supporting this, patients with i (9p) in fibroblasts tend to have more severe manifestations than those whose i (9p) is limited to lymphocytes, especially in terms of cardiac defects and viability (El Khattabi et al., 2015). Numerous data suggest that isochromosome 9p is predominantly present in peripheral blood but with a lower frequency in cell lines derived from the skin, amniotic fluid, or chorionic villus sampling. Hence, even a normal karyotype from amniocytes does not necessarily rule out the possibility of tetrasomy 9p (Cuoco et al., 1982; Papenhausen et al., 1990; Schaefer et al., 1991; Grass et al., 1993; Lloveras et al., 2004; McAuliffe et al., 2005). Additionally, the tissue-limited mosaicism mechanism at some extent unveiled that the prenatal cases of T9p showed numerous ultrasound abnormalities even with low-level mosaic status as the amniocytes are a mixture of cells derived from different germ layers (Chen et al., 2007). Based on published reports, non-mosaic fetuses have poorer prognosis, which explains the higher incidence of early death, FGR, Dandy-Walker malformation, and other congenital anomalies (Tang et al., 2004; Tan et al., 2007; El Khattabi et al., 2015). As such, the patient in the present study decided to terminate the pregnancy after genetic consultation.

Another factor that may affect the severity of the phenotype is breakpoint position; however, this remains controversial (Grass et al., 1993; Stumm et al., 1999; El Khattabi et al., 2015; Pinto et al., 2018; Vinksel et al., 2019). Some suggested that the phenotype will be more severe in patients that harbor the large portion of 9q extending to 9q21, which encompasses a large duplicated region with several OMIM morbid genes compared with those containing exclusively the entire 9p (El Khattabi et al., 2015; Pinto et al., 2018; Vinksel et al., 2019). A total of 21 prenatal cases with ultrasound anomalies have been reviewed and showed that involvement of the 9q region appears to have similar phenotypes to the p10 region in terms of facial anomalies, FGR, central nervous system dysfunction and cardiac anomalies (Table 1). Surprisingly, cardiac malformation seems to be much frequent when the region q12-q13 is involved.

**TABLE 1:** Malformations in relation to breakpoints among prenatal cases associated with T9p.

Breakpoints	Facial	FGR	CNS	Cardiac
p10 or p11 (*n* = 8)	4 (50%)	4 (50%)	4 (50%)	1 (12.5%)
McDowall et al. (1989); Schaefer et al. (1991); Dutly et al. (1998); Tang et al. (2004); Nakamura-Pereira et al. (2009); El Khattabi et al. (2015)				
q12 or q13 (*n* = 11)	6 (54.54%)	4 (36.36%)	6 (54.54%)	6 (54.54%)
Dhandha et al. (2002); Cazorla Calleja et al. (2003); Deurloo et al. (2004); Hengstschläger et al. (2004); Chen et al. (2007); di Vera et al. (2008); Lazebnik and Cohen, (2015); Wang et al. (2015); Vinkšel et al. (2019); Kok Kilic et al. (2022)				
q21 (*n* = 2)	1 (50%)	2 (100%)	0	0
Tan et al. (2007); Chen et al. (2014)				

Facial, facial dysmorphism including cleft/palate; FGR, fetal growth retardation; CNS, central nervous system dysfunction; Cardiac, cardiac anomalies.

Due to the poor resolution of conventional cytogenetic techniques, CMA with high throughput has been widely complemented into prenatal examinations for fetuses with ultrasound anomalies as it offers several advantages, such as fast reporting (without further cell culture) and high resolution in detecting copy number changes. Furthermore, it can provide a more precise location of the breakpoint of the tetrasomy 9p, thus improving the understanding of the genotype-phenotype correlation. However, the breakpoints of 9p10, q12, and q13 are located in the heterochromatin region, wherein a few markers have been set in CMA, making it hazardous to define the precise localization. With the release of the T2T-CHM13 reference genome, long-reads based whole genome sequencing may overcome the remaining gaps and provide convincing breakpoints for the complex chromosome rearrangement, including in T9p (Aganezov et al., 2022; Nurk et al., 2022).

T9p usually occurs *de novo*, and recurrence has been observed in only one report, indicating gonadal mosaicism (El Khattabi et al., 2015). Most supernumerary chromosomes are of maternal origin, including T9p (Kotzot et al., 1996; Dutly et al., 1998). The supernumerary chromosome can be separated into two forms according to the number of centromeres: i (9) with a single centromeric region or two centromeres forming idic (9). Several models have been proposed to explain the formation of an isochromosome. One mechanism relies on homologous recombination (HR) when intra-chromosomal U-type recombination occurs during meiosis I followed by non-disjunction in meiosis II (Floridia et al., 1996; Kotzot et al., 1996; Dutly et al., 1998; Knijnenburg et al., 2007). This U-type exchange is more likely to result in a dicentric than in a monocentric isochromosome. Another mechanism refers to the formation of monocentric isochromosomes and relies on centromere misdivision during the premeiotic stage followed by non-disjuntion at meiosis I (Rivera et al., 1986); alternatively, a non-disjuntion at meiosis II is followed by post-zygotic centromere misdivision (de Ravel et al., 2004). However, Kotzot et al. (1996) oppose to the former hypothesis in the formation of isochromosome 18p as this would require two abnormal cell divisions.

In conclusion, tetrasomy 9p is a rare chromosome rearrangement that often occurs *de novo*. A prenatal case of tetrasomy 9p without any ultrasound abnormality during the first trimester was revealed by NIPT and confirmed by invasive diagnosis. NIPT can screen not only for canonical trisomy 13, 18, and 21 but also large fragment copy number changes, such as tetrasomy 9p before it manifests with a significant phenotype. Furthermore, multiple techniques, such as karyotyping, FISH and CMA, are critical for a precise prenatal diagnostic.

## Data Availability

The datasets presented in this study can be found in online repositories. The names of the repository/repositories and accession number(s) can be found below: NCBI repository with the Accession number PRJNA880763, with the URL: https://dataview.ncbi.nlm.nih.gov/object/PRJNA880763.
